# Identification of Antileukemic Compound From Ginger by Integrating UHPLC–MS, Network Pharmacology, Molecular Docking, Molecular Dynamics Simulation, and In Vitro and In Vivo Experimental Validation

**DOI:** 10.1002/fsn3.72379

**Published:** 2026-09-16

**Authors:** Mengjun Huang, Jing Liu, Kaixuan Zeng, Lianling Xu, Yangchun Liu, Linyun Xie, Mengni Shi, Yan Zeng, Wenjun Liu, Ling Guo

**Affiliations:** ^1^ National‐Local Joint Engineering Research Center for Innovative Targeted Drugs Chongqing University of Arts and Sciences Chongqing P. R. China; ^2^ Department of Pediatric Hematologic Oncology and Respiratory Children's Medical Center, The Affiliated Hospital, Southwest Medical University, Sichuan Clinical Research Center for Birth Defects, Southwest Medical University Luzhou Sichuan P. R. China; ^3^ Pediatrics Department Southwest Medical University Luzhou Sichuan P. R. China; ^4^ Department of Clinical Medicine, School of Clinical Medicine Southwest Medical University Luzhou Sichuan P. R. China

**Keywords:** acute myeloid leukemia, arctigenin, ginger, molecular dynamics simulation, network pharmacology

## Abstract

Acute myeloid leukemia (AML) is a prevalent pediatric hematologic malignancy associated with a poor clinical prognosis, largely due to drug‐induced toxicity, the emergence of resistance, and frequent disease relapse. Edible plants offer promising complementary therapeutic approaches through their bioactive compounds, which are characterized by favorable safety profiles. *Zingiber officinale* (ginger), commonly consumed both as a dietary component and as a traditional medicinal herb, has garnered increasing scientific interest; however, its potential antileukemic properties remain largely unexplored. Crude extracts from four ginger cultivars exhibited antileukemic activity in AML cells. Metabolomic profiling was used to screen for candidate constituents, and Arctigenin was selected for further investigation. Its presence in the ginger extracts was verified by targeted UHPLC–MS analysis using an authentic standard, followed by evaluation of its antileukemic activity. Arctigenin showed potent and selective cytotoxicity against AML cells, particularly HL60 cells. Network pharmacology was employed to predict potential molecular targets, followed by molecular docking and 100‐ns molecular dynamics simulations to evaluate potential interactions, suggesting PARP1 and NFκB1 as potential molecular targets. Experimental analyses further demonstrated that Arctigenin induced apoptosis and altered NFκB and PARP protein expression in AML cells. In vivo experiments demonstrated that Arctigenin significantly suppressed tumor growth in an AML xenograft mouse model, achieving efficacy comparable to that of cytarabine, without inducing hepatosplenic toxicity or metabolic disturbances. These findings provide the first evidence supporting ginger as a novel phytochemical resource for AML therapy and support Arctigenin as a promising antileukemic candidate, underscoring its potential for incorporation into food‐based or plant‐derived therapeutic strategies.

## Graphical abstract

Metabolomic screening and UHPLC–MS verification identified Arctigenin as a candidate antileukemic compound from ginger. Multi‐dimensional computational and experimental validation demonstrated its anti‐AML activity through apoptosis induction and tumor growth suppression, with potential involvement of PARP1 and NFκB1.

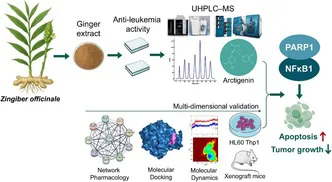

## 1 Introduction

Acute myeloid leukemia (AML) is a heterogeneous hematopoietic malignancy characterized by the uncontrolled proliferation of myeloid blasts, accounting for approximately 20% of pediatric leukemia cases (Xu et al. 2025). Despite advances in diagnostics, precise risk stratification, chemotherapy, and hematopoietic stem cell transplantation, survival outcomes in pediatric AML remain markedly inferior to those in acute lymphoid leukemia (ALL) (Tosic et al. 2023). This disparity is largely attributed to the high toxicity of chemotherapeutic agents (Wilson and Pemmaraju 2021), the emergence of drug resistance (Gangat and Tefferi 2025), and frequent relapse (Ganzel et al. 2018; Shimony et al. 2025). These persistent challenges highlight an urgent need for safer, less toxic, and more effective therapeutic strategies. Although interest in natural product–based therapies is growing, clinically approved antileukemic agents derived from natural sources remain limited, underscoring the untapped potential of this area.

Edible plants are widely recognized for their favorable safety profiles in therapeutic applications (Li et al. 2022; Zeng et al. 2024). Among them, ginger has been used both as food and medicine for over 5000 years, offering a rich source of bioactive compounds (Yin et al. 2018). The most studied components, curcumin and gingerols, have demonstrated diverse pharmacological activities. Curcumin is known for its anti‐inflammatory and anticancer properties, while gingerols exhibit antioxidant, anti‐inflammatory, and gastroprotective effects (Hong et al. 2020; Meng et al. 2024; Wu et al. 2023). These compounds are commonly employed as complementary treatments for metabolic syndromes, digestive disorders, and related conditions (Xiang et al. 2024). However, their clinical efficacy is limited by poor bioavailability and gastrointestinal irritation at higher doses (Gupta et al. 2025). Moreover, the therapeutic potential of many other ginger‐derived molecules remains poorly understood, particularly in the context of leukemia. In this study, we aim to systematically identify novel antileukemic agents from ginger and investigate their mechanisms of action at the molecular level.

In this study, we first evaluated the antileukemic activity of crude extracts from four ginger varieties to determine whether this bioactivity was consistently present across different cultivars. Metabolomic profiling was subsequently performed to screen for candidate constituents, through which Arctigenin was identified as a candidate for further investigation. Its presence in the ginger samples was subsequently verified using an authentic standard, and commercially available authentic Arctigenin was used for subsequent biological validation. To elucidate the underlying mechanisms, network pharmacology, molecular docking, and molecular dynamics simulations were integrated to identify potential molecular targets and characterize the interactions between Arctigenin and its candidate targets. Comprehensive in vitro and in vivo experiments were subsequently performed to further validate its antileukemic efficacy. Considering the Reduction principle of the 3Rs for animal welfare and the common practice in previous studies evaluating the antitumor activity of natural products in xenograft models, a single representative treatment dose was adopted for the in vivo study (Chen 2024; Chen et al. 2024; Liang et al. 2023; Sudhakaran et al. 2023; Yang et al. 2024). The selected dose of 100 mg/kg was based on previous studies demonstrating significant in vivo antitumor efficacy of Arctigenin together with no obvious chronic toxicity (Tan et al. 2018; Wang et al. 2017). These findings provide a solid experimental basis for the development of ginger‐derived antileukemic agents.

## 2 Materials and Methods

### 2.1 Cells and Reagents

The human acute myeloid leukemia (AML) cell lines Thp1 and HL60 and normal cell lines LO2, HUVEC, and HIBEC were obtained from the Cell Bank of the Chinese Academy of Sciences (Shanghai, China). AML and LO2 cells were cultured in RPMI‐1640 medium supplemented with 10% fetal bovine serum (FBS) and 1% penicillin–streptomycin, while HUVEC and HIBEC cells were cultured in DMEM under the same conditions (37°C, 5% CO_2_). The reagents used in this study included: RPMI‐1640, DMEM, FBS (Gibco, Grand Island, NY, USA), anti‐rabbit IgG‐HRP and anti‐mouse IgG‐HRP secondary antibodies (Shanghai, China), Cell Counting Kit‐8 (CCK‐8, Beyotime, Shanghai), apoptosis detection kit (BD Biosciences, San Diego, CA, USA), Bradford protein assay kit (Shanghai, China), and primary antibodies against GAPDH and β‐actin (Proteintech, Wuhan, China), as well as NFκB and cleaved PARP (Cell Signaling Technology, Danvers, MA, USA). Female BALB/c‐nu/nu mice (4–5 weeks old) were purchased from Huafukang Biotechnology Co. Ltd. (Beijing, China). A commercially available authentic Arctigenin standard (purity > 98%) was purchased from MedChemExpress (MCE, Shanghai, China) for subsequent biological validation.

### 2.2 Ginger Sample Collection and Preparation of Crude Extracts

Four ginger rhizome samples were selected: Luoping, Yunnan (L); tissue culture seedling from Luoping (P); Laiwu, Shandong (S); and tissue culture seedling from Laiwu (Z). For each type, 10 individual plants were collected to ensure representative sampling of each ginger cultivar from the respective regions. The rhizomes were thoroughly washed, placed in sterile bags, and transported to the laboratory in ice boxes maintained at 4°C. Upon arrival, samples were divided into small portions, rapidly frozen in liquid nitrogen, and stored at −80°C until further processing.

Based on the method described by Chen et al. (2013) with minor modifications, ginger rhizome samples were lyophilized and ground at 60 Hz for 1 min. Exactly 100 mg of the resulting powder was extracted with 500 μL of methanol–water (3:1, v/v) containing internal standards. After vortexing for 30 s, two stainless steel beads were added, and the mixture was homogenized at 45 Hz for 4 min, followed by sonication in an ice bath for 15 min. The suspension was then incubated on a mixer at 4°C overnight and centrifuged at 12,000 rpm for 15 min at 4°C. The supernatant was filtered through a 0.22 μm PTFE syringe filter, concentrated under reduced pressure at 60°C using a rotary evaporator, and finally lyophilized to obtain a dry ginger extract. The extract powder was dissolved in ddH_2_O to prepare different concentrations.

### 2.3 Cell Viability Assay

Cell viability was evaluated using the CCK‐8 kit, following the protocol described by Guo et al. (2021). Thp1 and HL60 cells were seeded into 96‐well plates at a density of 2 × 10^4^ cells/well and treated with serial concentrations of either crude extracts from four ginger varieties (0, 20, 40, 60, 80, and 100 μg/mL) or Arctigenin (0, 20, 40, 60, 80, and 100 μM). After 24, 48 and 72 h of incubation at 37°C with 5% CO_2_, 10 μL CCK‐8 solution was added to each well, followed by a further 4 h of incubation. Absorbance was measured at 450 nm using a microplate reader, and IC_50_ values were calculated using Graphpad Prism 9.5. The cytotoxicity of Arctigenin toward the normal human cell lines LO2, HUVEC, and HIBEC was evaluated under the same treatment conditions using the CCK‐8 assay.

### 2.4 Metabolomic Screening and Verification of Arctigenin

Metabolomic profiling of the four ginger samples was performed by Allwegene Technologies (Beijing, China) to screen for candidate constituents for subsequent investigation. Because Arctigenin identified during metabolomic screening is not generally recognized as a characteristic constituent of ginger, its presence in the four ginger samples was further verified using an authentic Arctigenin standard. Chromatographic separation was performed on a C18 column (50 × 4.6 mm, 3.5 μm) at 35°C using methanol–acetic acid (995:5, v/v) as the mobile phase at a flow rate of 0.3 mL/min, with an injection volume of 10 μL. Mass spectrometric detection was performed using a Q Exactive mass spectrometer (Thermo Fisher Scientific) equipped with an electrospray ionization (ESI) source operating in positive‐ion mode. The chromatographic retention behavior and mass‐spectrometric characteristics of the ginger samples were compared with those of the authentic Arctigenin standard for compound verification.

### 2.5 Network Pharmacology

#### 2.5.1 Prediction of Drug Targets for Anti‐AML Therapy

The potential targets of active compounds were predicted using SwissTargetPrediction (http://www.swisstargetprediction.ch/) and SuperPred (https://prediction.charite.de/). The resulting targets were standardized using the UniProt database (https://www.uniprot.org/), restricted to human proteins, and duplicates were removed. AML‐related genes were collected from the GeneCards (https://www.genecards.org/), OMIM (https://www.omim.org/), and PharmGKB (https://www.pharmgkb.org/) databases. After de‐duplication and UniProt validation, overlapping targets between compounds and disease were identified using R (v4.3.3), and visualized with the “VennDiagram” package.

#### 2.5.2 Protein–Protein Interaction (PPI) Network Construction

Intersecting targets were submitted to the STRING database (https://string‐db.org/) to generate a PPI network. Network topology was analyzed in Cytoscape using the cytoNCA plugin to identify core genes. A component–target–disease network was then constructed and visualized.

#### 2.5.3 GO and KEGG Enrichment Analysis

GO and KEGG pathway enrichment analyses were performed in R (version 4.3.3) based on intersecting drug–target genes. Results were visualized using the Bioinformatics online platform (http://www.bioinformatics.com.cn/).

#### 2.5.4 Molecular Docking

The 3D structures of active compounds were retrieved from PubChem (https://pubchem.ncbi.nlm.nih.gov/), and core target protein structures in PDB format were obtained from the Protein Data Bank (https://www.pdbus.org/). Docking simulations were performed using AutoDock Vina 1.5.7, and results were visualized and analyzed with PyMOL 2.6.0.

### 2.6 Molecular‐Dynamics Simulations

Molecular dynamics simulations of the protein–ligand complexes were performed using GROMACS 2022.5 with the CHARMM force field. Ligand parameters were generated via SwissParam (https://swissparam.ch). Systems were solvated with the TIP3P water model, maintaining at least 12 Å between solute and box edges. Neutralizing counter‐ions and 0.15 M NaCl were added to mimic physiological conditions. After energy minimization, temperature was gradually increased to 300 K over 200 ps, followed by a 2‐ns NPT equilibration at 1 bar. Production runs of 100 ns were conducted at 300 K and 1 bar with a 2 fs timestep. Van der Waals and short‐range electrostatic interactions used a 1.0 nm cutoff, and hydrogen bonds were constrained with the LINCS algorithm. Temperature and pressure were controlled by the V‐rescale thermostat and Berendsen barostat, respectively. Trajectory analyses included RMSD, RMSF, SASA, radius of gyration (Rg), and hydrogen bond number. Binding free energies were estimated via MM/PBSA using the last 20 ns of simulation data.

### 2.7 Apoptosis

Cells were seeded at 5 × 10^5^ cells/well in 6‐well plates and treated with Arctigenin at final concentrations of 0, 40, 60, and 80 μM for 24 h. After treatment, cells were harvested, washed twice with cold PBS, and resuspended in 500 μL Annexin V binding buffer. Annexin V‐FITC (5 μL) and propidium iodide (5 μL) were added, followed by incubation in the dark for 15 min. Apoptosis was assessed by flow cytometry using a BD FACSVerse cytometer (San Diego, CA, USA) (Guo et al. 2022).

### 2.8 Western Blotting

Treated cells were harvested, washed with PBS, and lysed using an ultrasonic cell disruptor. After centrifugation, the supernatant was collected as the protein extract. Protein concentrations were determined using a BCA assay kit, and equal amounts were subjected to SDS‐PAGE, followed by transfer onto PVDF membranes. Membranes were blocked with 7% skim milk for 1 h at room temperature and incubated overnight at 4°C with specific primary antibodies. After PBST washing, HRP‐conjugated secondary antibodies were added and incubated for 1 h at room temperature. Protein bands were visualized using a touch imaging system (E‐Blot Biotechnology, Shanghai, China). β‐actin and GAPDH served as internal controls for quantifying NFκB, PARP, and cleaved‐PARP expression, respectively (Guo et al. 2023).

### 2.9 In Vivo Animal Experiments

All animal procedures were approved by the Ethics Committee of Southwest Medical University (Protocol No. 20250305‐033). Female BALB/c‐nu/nu mice (4–5 weeks old, quality certificate number: B202504140383) were used to establish AML xenograft models. Mice were intraperitoneally injected with cyclophosphamide (2 mg/day, 200 μL) for two consecutive days to induce immunosuppression. Subsequently, 5 × 10^6^ HL60 cells were subcutaneously injected into the hind limb of each mouse. Once tumor volumes reached 50 ~ 100 mm^3^, mice were randomly divided into three groups (*n* = 6 per group): a negative control group (oral normal saline, 200 μL), a positive control group (intraperitoneal cytarabine, 5 mg/kg, 100 μL), and a treatment group (oral Arctigenin, 100 mg/kg, 200 μL). The experiment was terminated when the largest tumor volume approached 1500 mm^3^. Tumor volumes and body weights were recorded every 2 days. At the endpoint, tumors, livers, and spleens were excised and weighed for intergroup comparison. Tumor volume was calculated using the following formula:

$$\text{Tumor volume}\;\left({\mathrm{mm}}^{3}\right)=\frac{\text{length}\times {\text{width}}^{2}}{2}$$

Additionally, serum biochemical parameters were tested to evaluate hepatic and renal function, as well as systemic metabolic burden. At the end of the experiment, all animals were humanely euthanized (Xu et al. 2025).

### 2.10 Data Analysis

**FIGURE 1:**
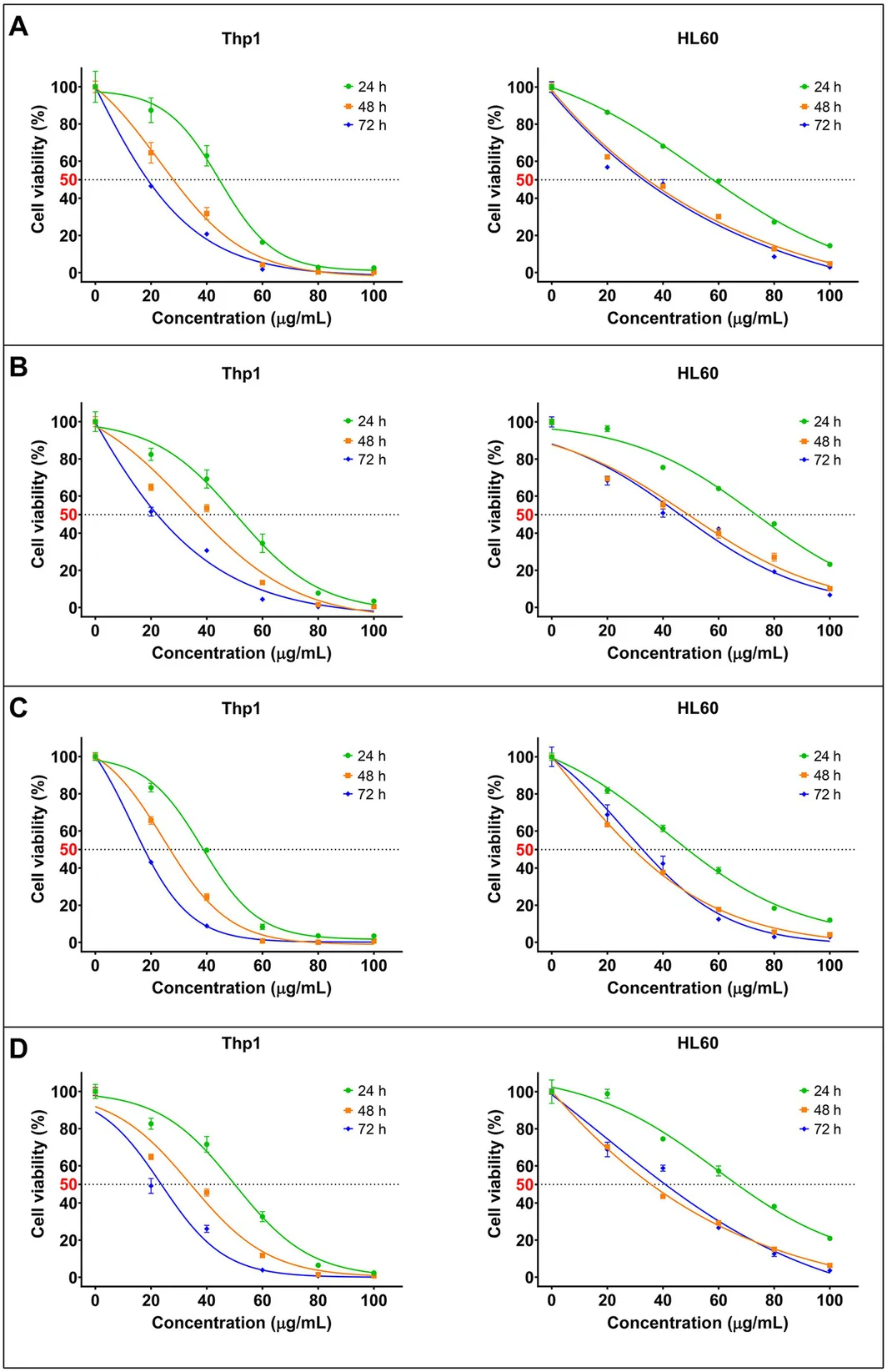
Effects of crude extracts from four ginger varieties on the viability of AML cell lines Thp1 and HL60. (A) Extract from variety L. (B) P. (C) S. (D) Z.

Statistical analyses were performed using one‐way ANOVA or unpaired *t*‐tests with GraphPad Prism 8.3.0.538. A *p*‐value < 0.05 was considered statistically significant. The Figures 1, 3 and 8 were analyzed using the CNSknowall platform (https://cnsknowall.com), a comprehensive web service for data analysis and visualization.

## 3 Results

### 3.1 Antileukemic Activity of Ginger Crude Extracts

Crude extract powders from four ginger varieties were tested at concentrations of 20, 40, 60, 80, and 100 μg/mL to evaluate their effects on the viability of two leukemia cell lines (HL60 and Thp1). As illustrated in Figure 1, the viability of both cell lines demonstrated a significant dose‐dependent decrease following treatments of 24, 48, and 72 h, respectively, indicating that all four ginger extracts effectively inhibited the proliferation of AML cells. Among the tested extracts, the S variety exhibited the lowest IC_50_ value of 27.82 ± 0.33 μg/mL against HL60 cells after 48 h of treatment, while the lowest IC_50_ value for Thp1 cells was 18.58 ± 0.23 μg/mL following a 72‐h treatment period (Table 1). Since all four ginger extracts exhibited antileukemic activity, metabolomic profiling was subsequently performed to screen for candidate constituents for further biological investigation.

**TABLE 1:** IC_50_ values of crude extracts from four ginger varieties after 24, 48 and 72 h of treatment.

Cultivar	Treatment time (h)	IC_50_ values (μg/mL)	
		HL60	Thp1
L	24	52.85 ± 0.07	40.38 ± 5.65
	48	30.93 ± 0.48	26.09 ± 2.76
	72	28.84 ± 0.87	20.1 ± 0.12
P	24	69.22 ± 0.80	42.1 ± 6.34
	48	39.71 ± 2.39	29.65 ± 0.78
	72	37.03 ± 2.25	22.51 ± 1.44
S	24	44.80 ± 1.95	35.14 ± 2.11
	48	27.82 ± 0.33	25.08 ± 0.93
	72	29.76 ± 3.84	18.58 ± 0.23
Z	24	63.85 ± 0.65	43.68 ± 4.04
	48	33.43 ± 0.86	28.99 ± 0.85
	72	35.64 ± 2.86	21.24 ± 2.30

### 3.2 Metabolomic Screening and Verification of Arctigenin in Ginger

**FIGURE 2:**
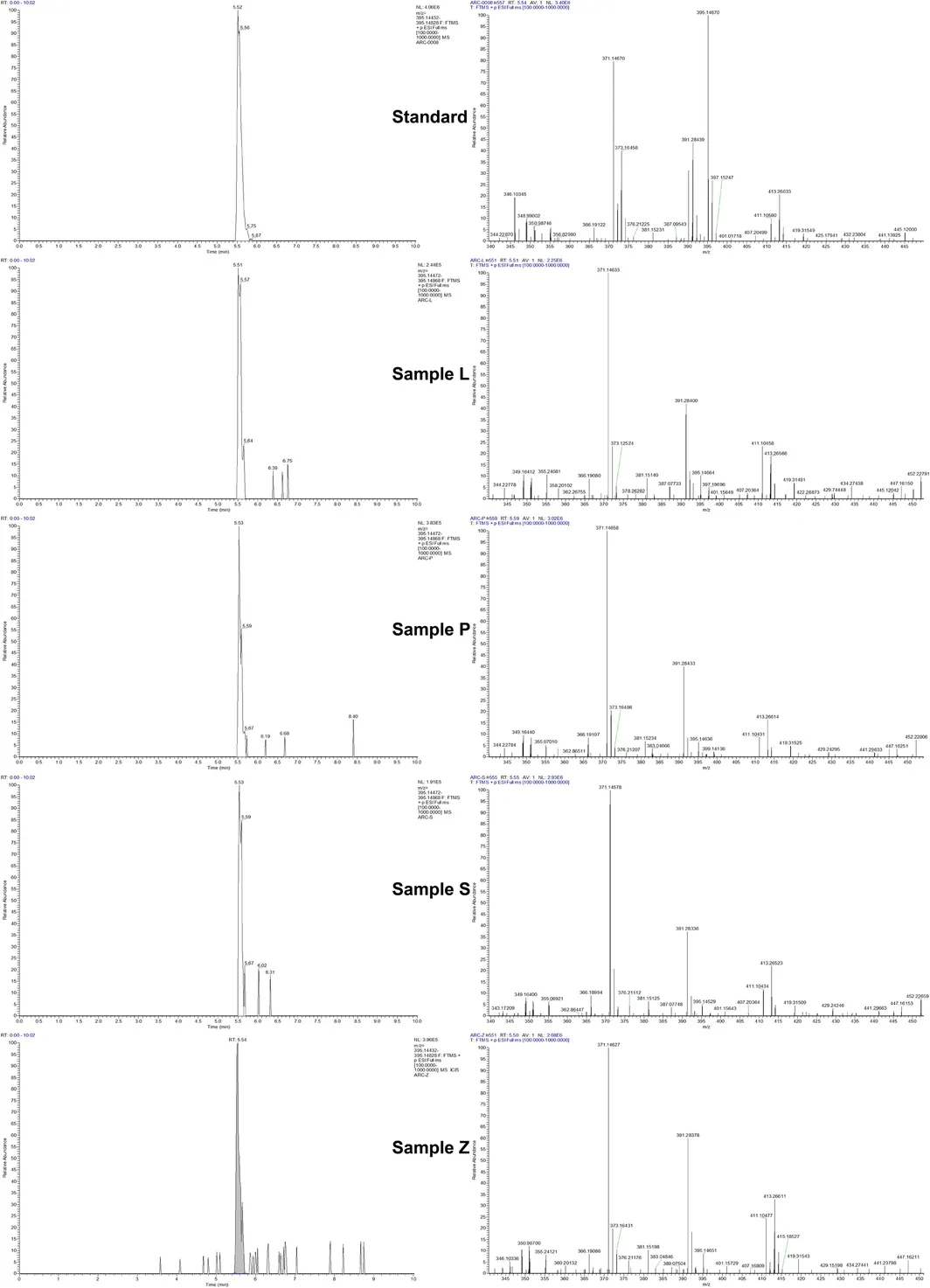
Validation of Arctigenin in ginger samples by UHPLC–MS analysis using an authentic standard. Representative chromatograms and mass spectra of the authentic Arctigenin standard and ginger extracts from four cultivars (L, P, S, and Z) are shown.

Metabolomic profiling of the four ginger samples was performed as a screening step to identify candidate constituents for further investigation. Among the candidate compounds, Arctigenin was consistently detected across all four ginger cultivars and was selected for further evaluation based on its reported antitumor activities. Targeted UHPLC–MS analysis using an authentic Arctigenin standard was subsequently performed to verify its presence in the ginger samples. As shown in Figure 2, the authentic Arctigenin standard exhibited a characteristic chromatographic peak at approximately 5.52–5.54 min, whereas the corresponding peaks in the L, P, S, and Z ginger samples were detected at 5.51, 5.53, 5.53, and 5.54 min, respectively. Mass‐spectrometric analysis in positive‐ion mode further showed the characteristic sodium‐adduct ion of Arctigenin ([M + Na]^+^, m/z 395) in the ginger samples, consistent with that of the authentic standard. The close agreement in both chromatographic retention behavior and characteristic mass‐spectrometric signals between the ginger samples and the authentic standard provides further evidence supporting the presence of Arctigenin in all four ginger samples examined in this study. A commercially available authentic Arctigenin standard was therefore used for all subsequent biological experiments.

### 3.3 AntiLeukemic Activity and Selectivity of Arctigenin

**FIGURE 3:**
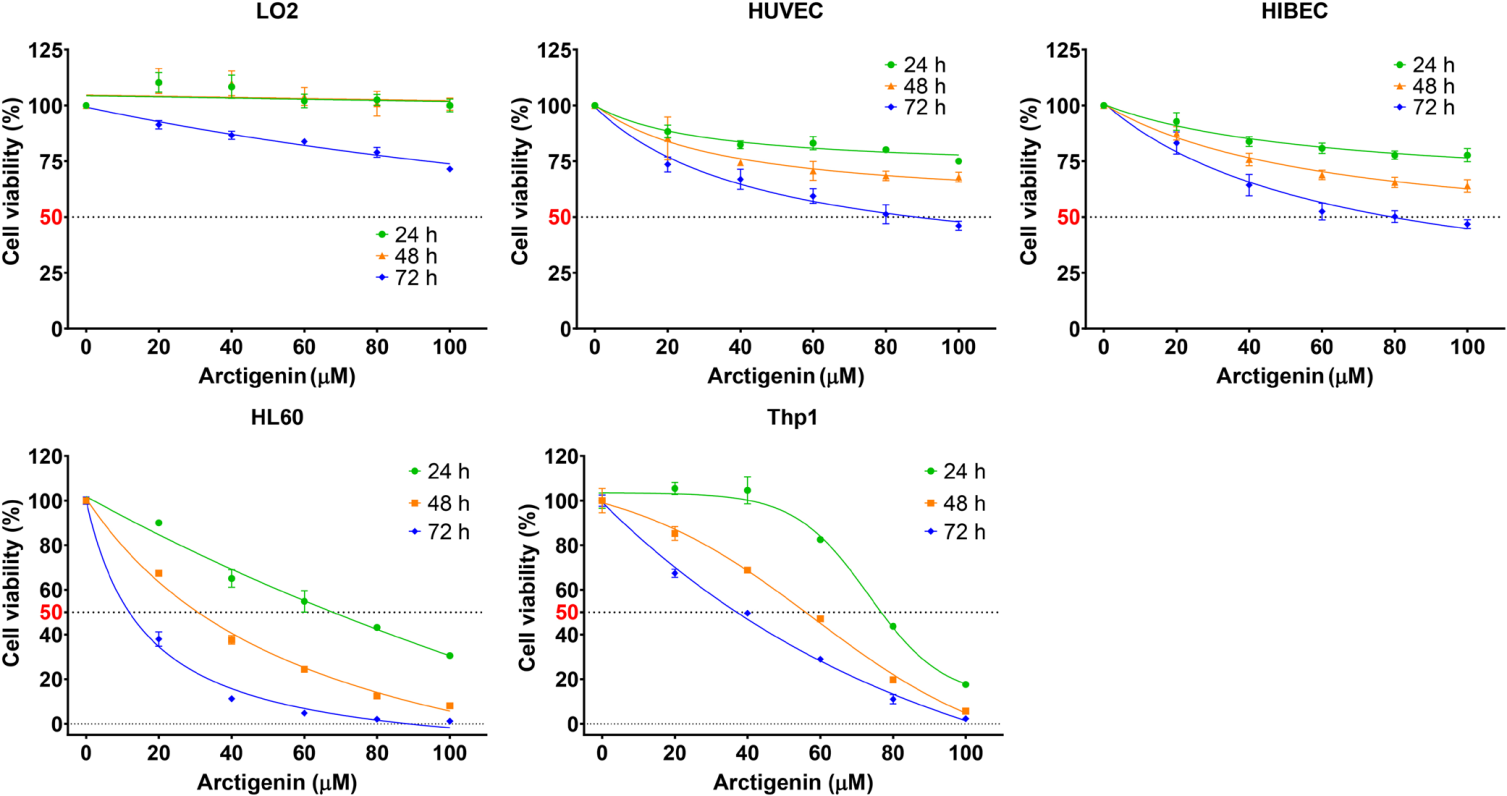
Effects of Arctigenin on the viability of AML cell lines and normal cells.

The antileukemic activity and selectivity of Arctigenin were further evaluated in AML and normal cells. Arctigenin inhibited the viability of HL60 and Thp1 cells in a dose‐ and time‐dependent manner (Figure 3). The IC_50_ values for HL60 cells were 64.58 ± 5.84, 30.20 ± 1.46, and 16.27 ± 1.97 μM at 24, 48, and 72 h, respectively, whereas those for Thp1 cells were 77.21 ± 0.52, 49.07 ± 2.34, and 33.97 ± 0.82 μM, respectively (Table 2).

**TABLE 2:** IC_50_ values and therapeutic index of Arctigenin after 24, 48 and 72 h of treatment.

Cell lines		IC_50_ values (μM)		
		Treatment time (h)		
		24	48	72
AML cells	HL60	64.58 ± 5.84	30.20 ± 1.46	16.27 ± 1.97
	Thp1	77.21 ± 0.52	49.07 ± 2.34	33.97 ± 0.82
Normal cells	LO2	> 100	> 100	> 100
	HUVEC	> 100	> 100	89.56 ± 6.65
	HIBEC	> 100	> 100	82.87 ± 11.16
Normal cells	AML cells	Therapeutic index (TI) = $\frac{{\mathrm{IC}}_{50,\;\text{normal cells}}}{{\mathrm{IC}}_{50,\mathrm{AML}\;\text{cells}}}$		
LO2	HL60	> 1.55	> 3.31	> 6.15
HUVEC				5.50
HIBEC				5.09
LO2	Thp1	> 1.30	> 2.04	> 2.94
HUVEC				2.64
HIBEC				2.44

*Note:* When the IC_50_ in normal cells exceeded the highest tested concentration (100 μM), the corresponding TI is reported as a lower‐bound value (>).

In contrast, Arctigenin showed substantially lower cytotoxicity toward the normal cell lines LO2, HUVEC, and HIBEC (Figure 3). The IC_50_ values for all three normal cell lines were > 100 μM at 24 and 48 h. At 72 h, the IC_50_ remained > 100 μM for LO2 cells and was 89.56 ± 6.65 and 82.87 ± 11.16 μM for HUVEC and HIBEC cells, respectively. Accordingly, the therapeutic index (TI), calculated as IC_50_(normal cells)/IC_50_ (AML cells), ranged from > 1.30 to > 6.15 or 2.44–5.50, depending on the cell line and treatment duration (Table 2). These results indicate that Arctigenin exhibits greater cytotoxicity toward AML cells than toward the tested normal cells, supporting its selective antileukemic activity.

### 3.4 Network Pharmacology Analysis

#### 3.4.1 Arctigenin and AML‐Related Genes

**FIGURE 4:**
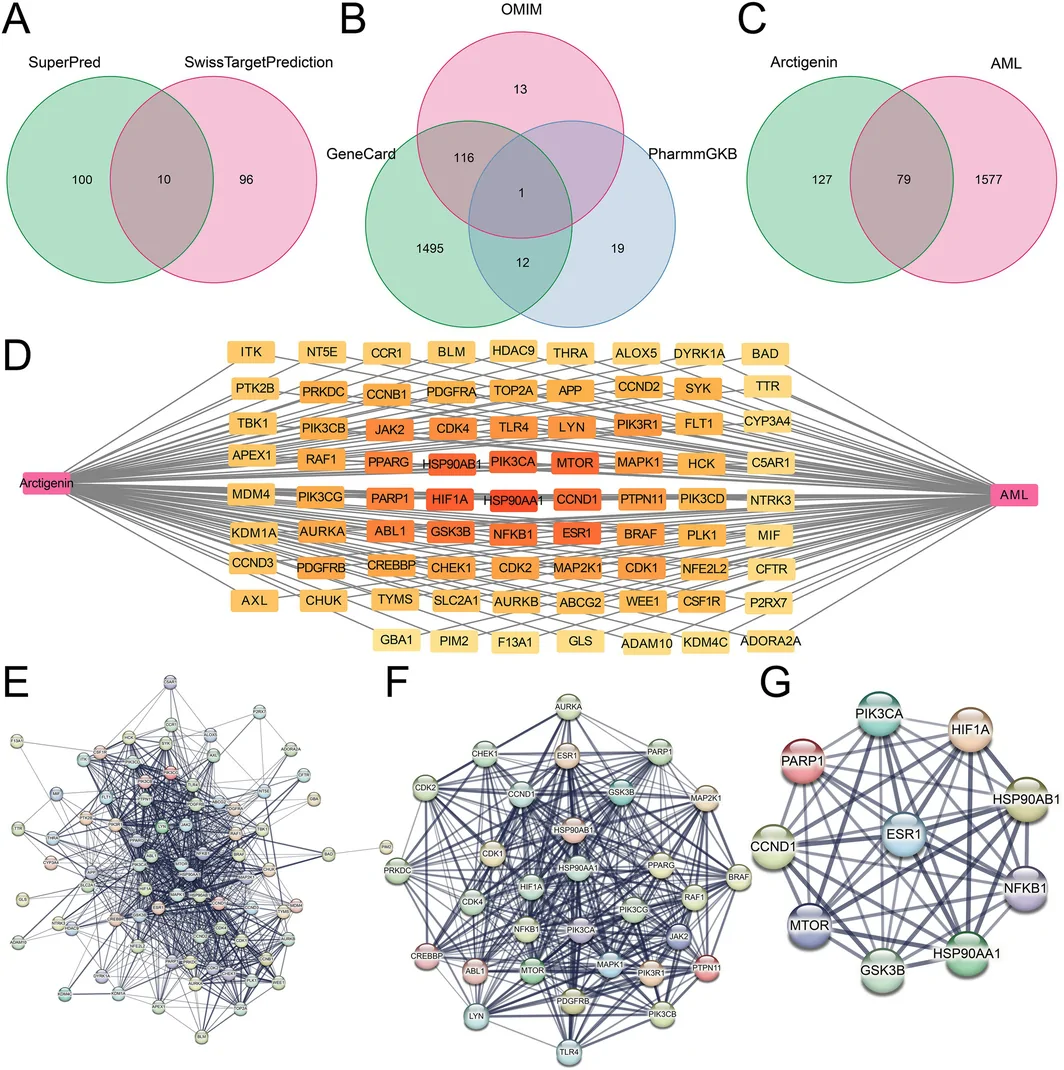
Predicted molecular targets of Arctigenin against AML. (A) Potential targets of Arctigenin. (B) Venn diagram of AML‐related genes from three databases. (C) Overlapping genes between Arctigenin targets and AML‐related genes. (D) Compound–target–disease network of Arctigenin in AML. (E) PPI network of overlapping AML‐related target genes. (F) Further screening of key AML‐related targets of Arctigenin. (G) 10 hub genes identified as candidate targets of Arctigenin against AML.

A total of 206 potential targets of Arctigenin were identified through comprehensive screening of the SwissTargetPrediction and SuperPred databases (Figure 4A). AML‐related targets were collected from GeneCards (15,913 genes), OMIM (130 genes), and PharmGKB (32 genes). GeneCards entries were filtered using a relevance score > 10, resulting in 1624 candidate genes (Figure 4B). After merging entries from all three databases, 1656 AML‐related genes were obtained. Intersection analysis revealed 79 overlapping genes between the predicted targets of Arctigenin and AML‐related genes (Figure 4C), suggesting these as potential therapeutic targets of Arctigenin in AML.

#### 3.4.2 Core Targets and Network Interactions

A tripartite disease–drug–target network was constructed (Figure 4D). Topological analysis of the protein–protein interaction (PPI) network using the CytoNCA plugin identified 10 core genes based on centrality parameters: NFκB1, CCND1, GSK3B, HSP90AA1, PARP1, ESR1, HIF1A, PIK3CA, MTOR, and HSP90AB1 (Figure 4E–G).

#### 3.4.3 GO and KEGG Enrichment Analysis

**FIGURE 5:**
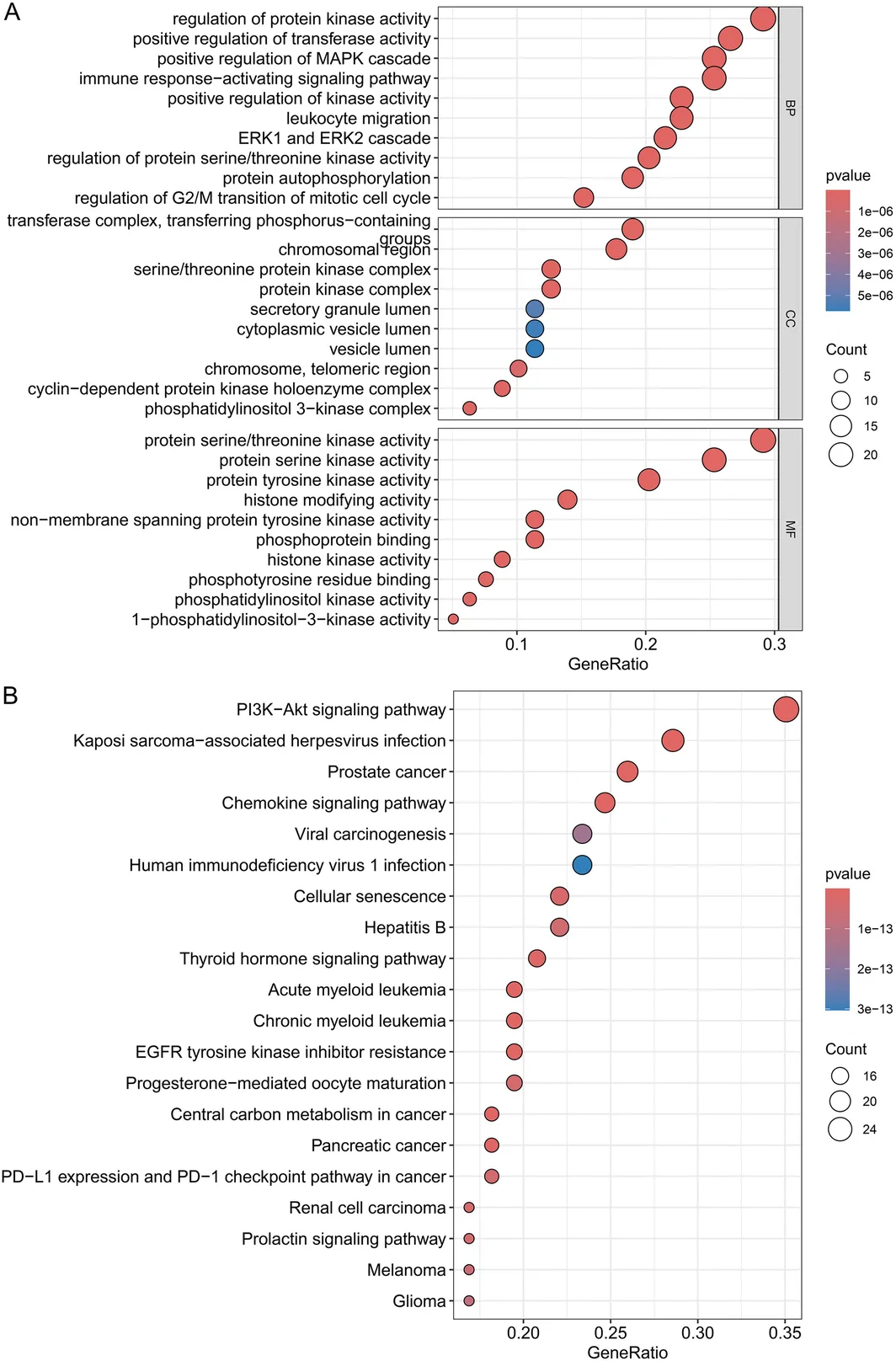
Enrichment analysis. (A) GO enrichment analysis. (B) KEGG pathway enrichment analysis.

Enrichment analysis identified biological processes and signaling pathways potentially associated with the anti‐AML activity of Arctigenin. GO analysis indicated that the predicted targets of Arctigenin were primarily associated with the regulation of protein kinase activity, including MAPK signaling and serine/threonine and tyrosine kinase functions (Figure 5A). KEGG pathway analysis showed significant enrichment of the predicted targets in AML‐ and CML‐related pathways, with notable enrichment also observed in the PI3K‐Akt signaling pathway, which is closely associated with leukemia cell survival (Figure 5B).

#### 3.4.4 Molecular Docking

**FIGURE 6:**
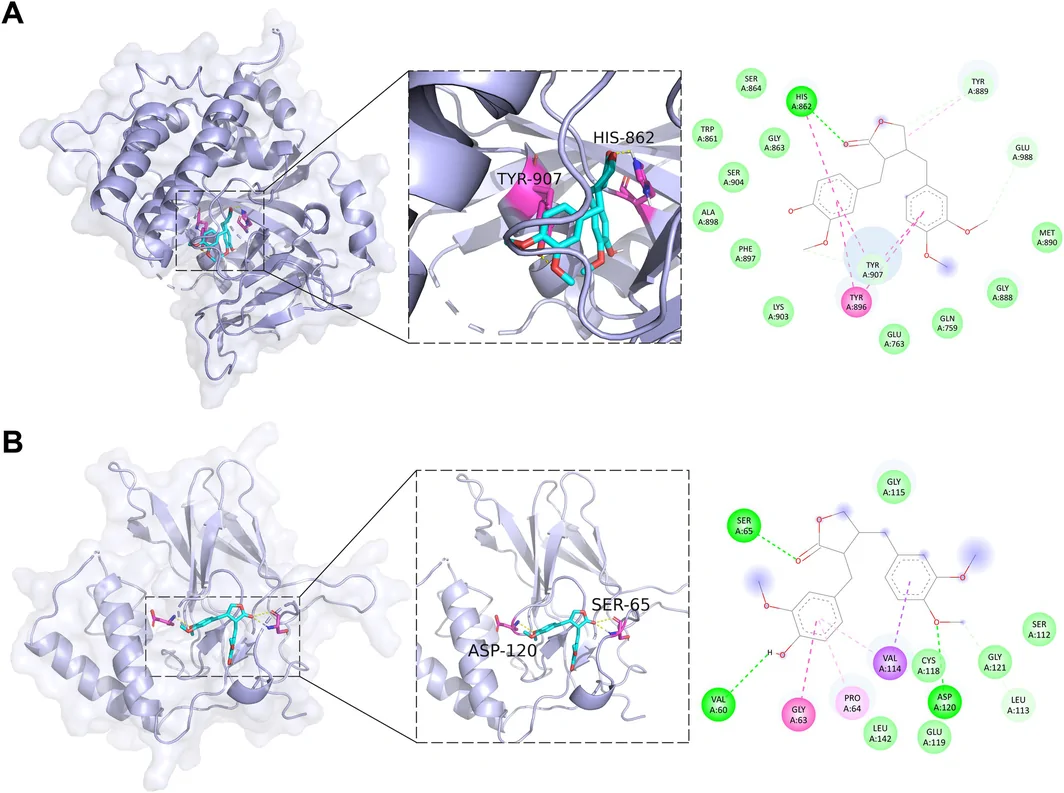
Molecular docking of Arctigenin with hub targets. (A) PARP1. (B) NFκB1. Structures were visualized and analyzed using PyMOL and Discovery Studio 2019 Client. Proteins are shown in light blue, Arctigenin in cyan, and interacting residues highlighted in magenta.

To further investigate the predicted therapeutic targets, we examined the expression profiles of core genes identified through PPI topological analysis using the GEPIA and Human Protein Atlas databases. Notably, the key inflammation‐ and apoptosis‐related regulators NFκB1 and PARP1 exhibited differential expression at both mRNA and protein levels in AML (Figures S1 and S2). Molecular docking was subsequently conducted to assess the binding interactions between Arctigenin and these targets. The results revealed favorable binding affinities, with docking scores of −8.7 kcal/mol for PARP1 and −6.3 kcal/mol for NFκB1. Both targets formed multiple hydrogen bonds with Arctigenin, suggesting stable ligand–receptor interactions (Figures 6 and S3).

### 3.5 Molecular Dynamics Simulation

**FIGURE 7:**
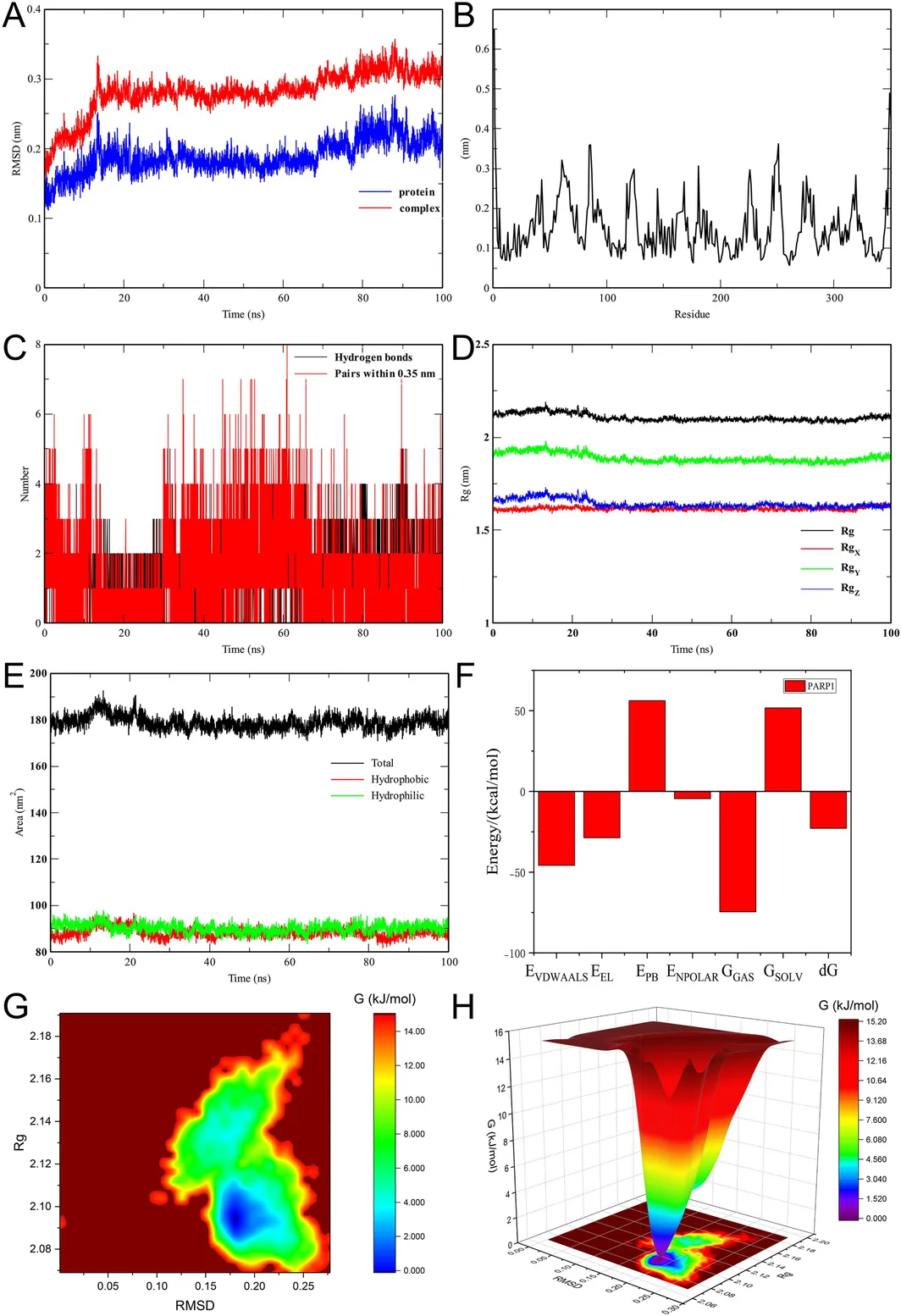
Molecular dynamics simulation (MDS) results for the Arctigenin‐PARP1 complex. (A) RMSD values over simulation time. (B) Protein flexibility variations (RMSF) during simulation. (C) Hydrogen bond dynamics throughout the simulation. (D) Radius of gyration (Rg) curve. (E) Solvent‐accessible surface area (SASA) changes. (F) MMPBSA binding free energy analysis. (G, H) Two‐dimensional and three‐dimensional free energy landscape mappings.

To assess the dynamic stability of Arctigenin–target interactions, molecular dynamics (MD) simulations were performed. Arctigenin exhibited more stable binding with PARP1 than with NFκB1 (Figures 7 and S4). The Arctigenin–PARP1 complex stabilized after 15 ns with RMSD < 0.1 nm, whereas the NFκB1 complex showed moderate stability with RMSD < 0.2 nm from 25 to 60 ns (Figure 7A, S4A). Residue‐level analysis revealed higher flexibility in NFκB1 (residues 74–79, 176–180, 184–187, 222–230), while PARP1 maintained consistently low RMSF values, indicating greater rigidity (Figures 7B and S4B). Hydrogen bond analysis further supported these findings: Arctigenin–PARP1 retained numerous hydrogen bonds throughout the simulation, whereas bond loss in the NFκB1 complex correlated with increased RMSD and partial dissociation (Figures 7C and S4C). Both complexes maintained stable global conformations (Rg and SASA), though Arctigenin–NFκB1 exhibited a larger Rg, reflecting looser binding (Figures 7D,E and S4D,E). MMPBSA analysis confirmed strong binding affinity of Arctigenin to PARP1 (ΔG = −22.82 kcal/mol), mainly driven by van der Waals (−45.82 kcal/mol) and electrostatic interactions (−28.74 kcal/mol), partially offset by unfavorable polar solvation energy (+56.23 kcal/mol) (Figure 7F). Finally, Gibbs free energy landscapes (GELs) revealed a stable energy basin for Arctigenin–PARP1 (Rg = 2.08–2.11 nm, RMSD = 0.16–0.22 nm), with two adjacent minima suggesting dynamic equilibrium. In contrast, the NFκB1 complex featured a higher‐energy metastable state (Figures 7G,H and S4G,H).

### 3.6 Western Blotting

**FIGURE 8:**
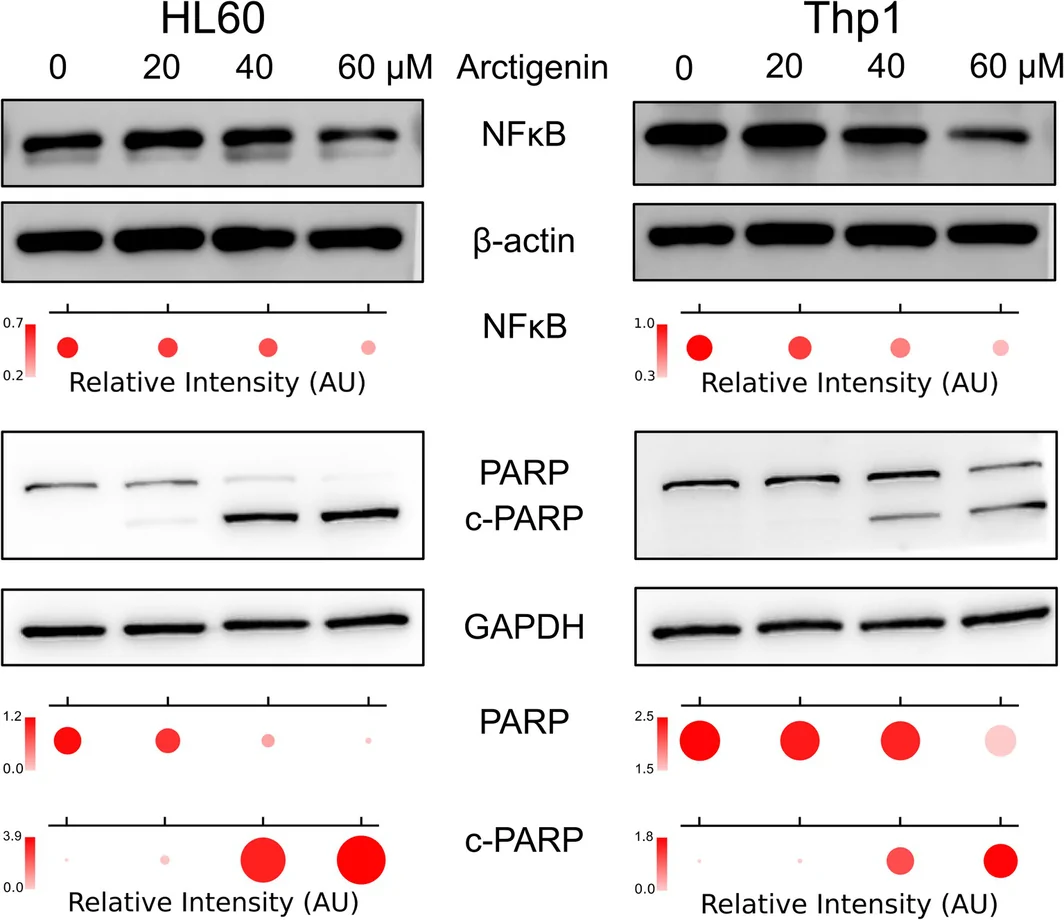
Western blot analysis of NFκB, PARP, and c‐PARP expression in HL60 and Thp1 cells treated with varying concentrations of Arctigenin.

Western blotting was performed to examine the effects of Arctigenin on NFκB and PARP expression in HL60 and Thp1 cells. As shown in Figure 8, Arctigenin treatment reduced NFκB protein expression, with a more pronounced decrease observed at higher concentrations. Meanwhile, Arctigenin altered PARP expression and cleavage, as evidenced by a reduction in PARP accompanied by a marked increase in cleaved PARP (c‐PARP), particularly in HL60 cells. These findings demonstrate that Arctigenin affects both NFκB and PARP expression in AML cells.

### 3.7 Apoptosis

**FIGURE 9:**
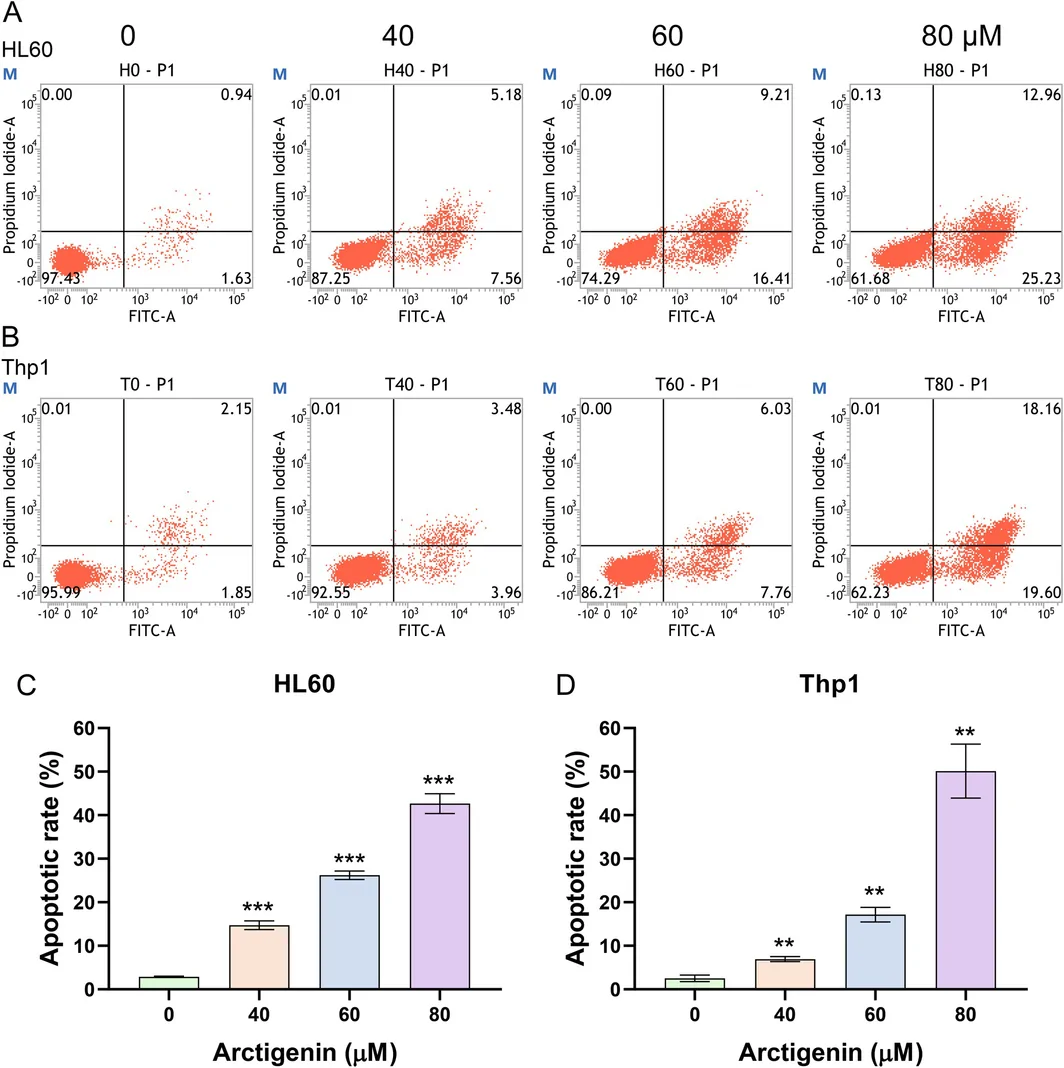
Arctigenin induces apoptosis in AML cells. (A) HL60. (B) Thp1. (C, D) Statistical analysis of apoptosis rates of HL60 and Thp1. (**p < 0.01, ***p < 0.001 compared to the control).

To experimentally evaluate the pro‐apoptotic effects of Arctigenin predicted by network pharmacology, apoptosis in HL60 and Thp1 cells was quantified by flow cytometry following treatment with 40, 60, and 80 μM Arctigenin. As shown in Figure 9, Arctigenin significantly increased both early and late apoptotic populations in a dose‐dependent manner, confirming its pro‐apoptotic activity. Notably, HL60 cells exhibited markedly greater sensitivity than Thp1 cells, with higher total apoptosis rates at equivalent doses, especially at 40 and 60 μM, consistent with prior cell viability findings.

### 3.8 In Vivo Experiments

**FIGURE 10:**
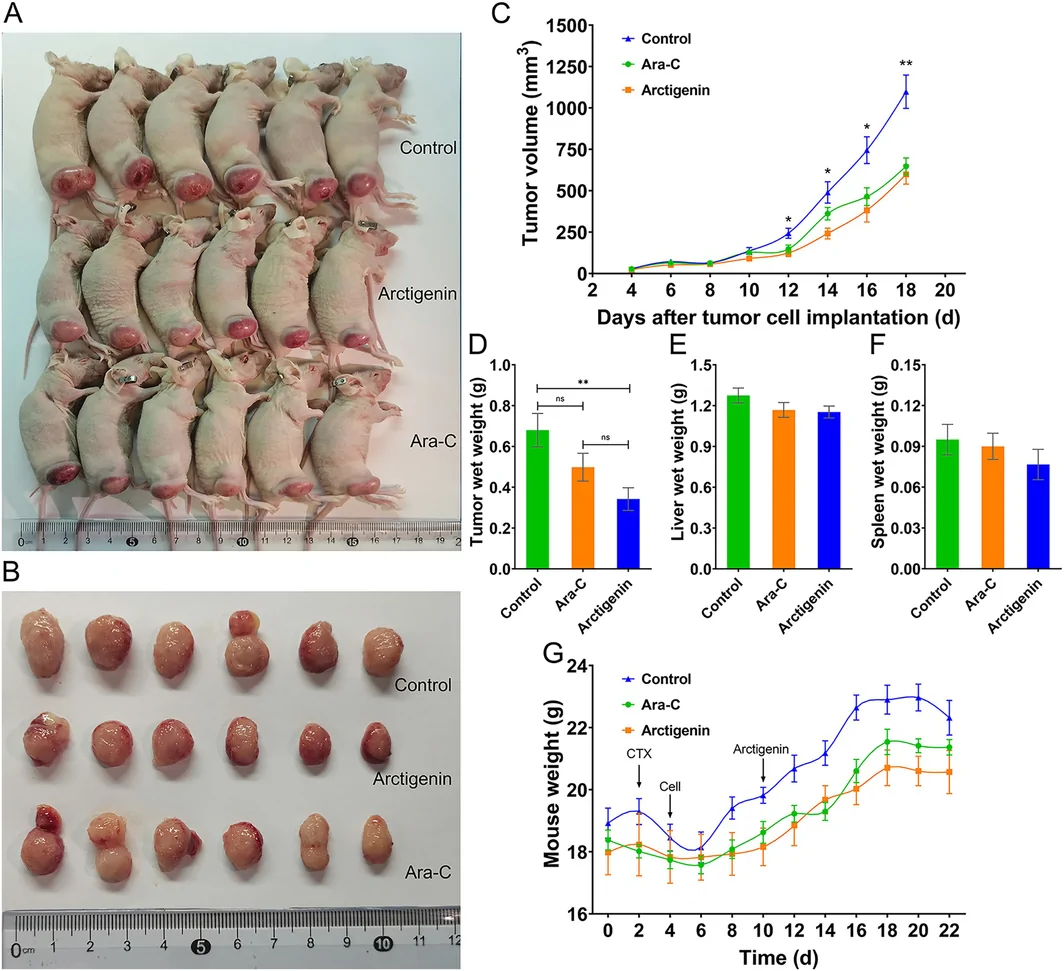
In vivo anti‐AML effects of Arctigenin in xenograft models. (A) Xenograft models. (B) Tumors. (C) Tumor volume changes over time. (D) Final tumor weights. (E, F) Liver and spleen weights. (G) Body weight monitoring of mice during treatment.

To further assess the antileukemic effect of Arctigenin, an AML xenograft mouse model was established with three groups: control, cytarabine (Ara‐C) positive control, and Arctigenin treatment. Tumors formed in the right hind limb (Figure 10A) were largest in the control group, while Arctigenin and Ara‐C treatment significantly reduced tumor size. Excised tumor comparison revealed larger, irregular masses in controls, whereas Arctigenin‐ and Ara‐C‐treated tumors were smaller and more uniform, confirming antitumor activity (Figure 10B). Longitudinal monitoring (Figure 10C) showed markedly slower tumor growth in the Arctigenin group, comparable to Ara‐C. Final tumor weights were significantly lower in both treatment groups versus controls (*p* < 0.01), without significant difference between Arctigenin and Ara‐C, indicating similar therapeutic efficacy (Figure 10D). As shown in Figure 10E,F, liver and spleen weights showed no significant changes among groups, suggesting no obvious hepatosplenic toxicity under the experimental conditions. Serum biochemistry (Table 3) further indicated no evidence of hepatorenal impairment or metabolic dysfunction in Arctigenin‐treated mice, supporting a favorable preliminary safety profile under the experimental conditions. Although early weight loss was observed due to tumor burden and treatment stress (Figure 10G), both Arctigenin‐ and Ara‐C‐treated mice recovered in later stages, reflecting good overall tolerability.

**TABLE 3:** Serum biochemical analysis in AML xenograft mice.

	Control	Ara‐C	Arctigenin
ALT	61.96 ± 13.26	55.18 ± 8.87	59.59 ± 34.22
AST	194.52 ± 48.48	173.64 ± 12.61	206.45 ± 66.62
AST/ALT	3.56 ± 0.83	3.71 ± 0.85	4.88 ± 2.53
TP	50.83 ± 0.88	49.78 ± 1.10	49.89 ± 1.50
ALB	30.42 ± 1.27	30.55 ± 0.86	29.87 ± 1.08
GLO	20.42 ± 0.89	19.23 ± 0.31	20.02 ± 1.10
A/G	1.70 ± 0.34	1.85 ± 0.39	1.83 ± 0.40
LDH	2368.37 ± 930.71	2299.23 ± 630.52	2425.12 ± 590.11
Urea	8.40 ± 1.52	7.59 ± 0.82	8.33 ± 1.41
UA	255.25 ± 105.43	269.38 ± 90.48	208.07 ± 52.25
Crea	14.81 ± 2.21	13.92 ± 2.91	11.77 ± 1.44
GFR	276.16 ± 86.82	292.38 ± 96.88	327.28 ± 102.35

## 4 Discussion

This study aimed to identify novel, low‐toxicity bioactive compounds from ginger with potent antileukemic activity and to elucidate their underlying mechanisms. Ginger, a widely consumed medicinal and culinary plant, offers an attractive therapeutic source due to its food‐grade safety and long‐standing use in traditional medicine (Ali et al. 2008). Among its phytochemicals, curcumin and gingerols have been the most extensively studied. However, the clinical application of curcumin and gingerols is hampered by their poor bioavailability and dose‐dependent gastrointestinal toxicity. Current efforts predominantly focus on structural modification or advanced delivery systems to mitigate these limitations (Mallya and Lewis 2025). In contrast, other constituents of ginger remain largely unexplored, particularly with regard to their potential antileukemic effects. Our work thus sought to uncover previously unrecognized antileukemic agents from ginger and clarify their molecular targets and pathways.

This study demonstrated that crude extracts from all four tested ginger varieties exerted significant, dose‐ and time‐dependent inhibitory effects on the viability of HL60 and Thp1 AML cell lines. These consistent results across the four ginger samples suggest that antileukemic activity may be shared across different ginger sources rather than being restricted to a single sample or cultivar. This preliminary evidence supports the potential of ginger as a broadly applicable natural resource for developing novel therapeutic or adjunctive agents against AML.

Following confirmation of the antileukemic activity of the ginger extracts, metabolomic profiling was performed to screen for candidate constituents for further investigation, through which Arctigenin was identified for further evaluation. Arctigenin is a lignan that is not generally regarded as a canonical or characteristic constituent of ginger. Therefore, its presence in the ginger samples was not inferred solely from the metabolomic screening results. Comparison with an authentic Arctigenin standard showed closely matched chromatographic retention behavior and characteristic mass‐spectrometric signals, supporting the presence of Arctigenin in all four ginger samples examined in this study (Figure 2). Nevertheless, the present study did not perform validated absolute quantification of Arctigenin in ginger; therefore, no conclusion is made regarding its absolute abundance or whether it represents a major constituent of ginger. Given the reported antitumor potential of lignans (Wang, Qin, et al. 2023), Arctigenin was selected for further investigation. Its dibenzyl butyrolactone skeleton and chiral lactone center have been associated with its anti‐inflammatory, antioxidant, and anticancer activities (Sólyomváry et al. 2015; Wang, Ge, et al. 2023). However, its direct antileukemic activity has not been systematically evaluated. In the present study, Arctigenin demonstrated substantial anti‐AML activity against both HL60 and Thp1 cells, supporting its selection as a candidate bioactive constituent from ginger. HL60 cells were more sensitive at lower concentrations, whereas higher concentrations effectively suppressed viability in both cell lines (Figure 3). This differential sensitivity may reflect the biological heterogeneity of AML and warrants further mechanistic investigation.

To explore the potential molecular basis of Arctigenin's antileukemic activity, network pharmacology analysis was performed to identify candidate targets for further investigation. Ten core genes were identified through PPI topological analysis. Among these, only CCND1, HIF1A, NFκB1, HSP90AB1, and PARP1 were significantly differentially expressed between AML patients and healthy controls (Figure S1). Further analysis demonstrated that HSP90AB1, CCND1, and HIF1A exhibited only low‐level RNA or protein expression in leukemia. In contrast, NFκB1 and PARP1 displayed coordinated high expression at both RNA and protein levels in leukemia (Figure S2). Therefore, NFκB1 and PARP1 were selected as candidate targets for further computational investigation, and molecular docking was subsequently performed to evaluate their potential interactions with Arctigenin.

Molecular docking revealed a strong binding affinity between Arctigenin and PARP1 (binding energy = −8.7 kcal/mol), primarily mediated by hydrogen bonds and van der Waals interactions involving residues Glu988, Gly863, Lys903, Ser904, and Tyr907 (Figure 6A). Notably, Glu988, Gly863, Tyr907, and Lys903 are functionally critical for PARP1's roles in DNA damage repair and transcriptional regulation (Nilov et al. 2020; Pushkarev et al. 2024), with Tyr907, Gly863, and Ser904 overlapping with known inhibitor‐binding sites (Figure S3) (Johannes et al. 2024). These findings suggest a potential molecular interaction between Arctigenin and PARP1 involving functionally relevant residues. In comparison, Arctigenin exhibited a moderate binding affinity to NFκB1 (binding energy = −6.3 kcal/mol), interacting with residues Pro64, Gly63, Glu62, Val60, and Cys61, some of which are associated with functional activity (Takahashi et al. 2024). These results indicated both proteins may be potential targets of Arctigenin, with a stronger interaction observed for PARP1. To further evaluate binding stability and dynamic behavior, we conducted MD simulations. The Arctigenin‐PARP1 complex reached dynamic equilibrium within ~15 ns and remained stable throughout the simulation, supported by persistent hydrogen bonding (Figure 7). The average binding free energy (−22.82 kcal/mol) further confirmed the stability and affinity of the complex. Conversely, although the Arctigenin‐NFκB1 complex initially reached a relatively stable conformation, it failed to maintain the stability over the entire 100 ns simulation (Figure S4). These dynamic simulations corroborated the molecular docking results, indicating that PARP1 and NFκB1 are potential targets of Arctigenin. Of the two, PARP1 appears to be more significantly affected by Arctigenin than NFκB1.

Subsequent Western blot analysis provided further experimental support for the potential involvement of PARP1 and NFκB1 in the response to Arctigenin. Notably, Arctigenin affected PARP expression while promoting its cleavage into c‐PARP, supporting the involvement of PARP‐associated changes in Arctigenin‐induced apoptosis. Meanwhile, Arctigenin also reduced NFκB protein expression, particularly at higher concentrations (Figure 8). Together with the network pharmacology, molecular docking, and MD simulation results, these findings suggest that PARP1 and NFκB1 may participate in the antileukemic effects of Arctigenin. Importantly, both PARP1 and NFκB1 are recognized therapeutic targets in leukemia (Di Francesco et al. 2022; Padella et al. 2022). Moreover, given their close association with apoptosis pathways (Ke et al. 2022), apoptosis induction may contribute to the antileukemic effects of Arctigenin. As shown in Figure 9, Arctigenin robustly induced apoptosis in AML cells. Flow cytometry of HL60 and Thp1 cells revealed a clear dose‐dependent increase in apoptosis, with cell line‐specific sensitivity patterns consistent with prior viability assays, further supporting both the experimental findings and the predictions derived from network pharmacology.

Finally, in vivo validation using AML xenograft models demonstrated that Arctigenin significantly suppressed tumor growth, with efficacy comparable to the clinical chemotherapeutic agent Ara‐C (Guinan et al. 2020; Yoon et al. 2019). Arctigenin treatment led to reduced tumor volume and weight (Figure 10), confirming its antileukemic activity in vivo, and aligning with previous cellular findings. More importantly, Arctigenin exhibited a favorable safety profile. In vitro, Arctigenin showed substantially lower cytotoxicity toward the normal cell lines LO2, HUVEC, and HIBEC than toward AML cells (Figure 3). The calculated therapeutic index further supported the preferential cytotoxicity of Arctigenin toward AML cells, particularly HL60 cells. In vivo, Arctigenin caused no hepatosplenic damage or abnormal serum biochemical changes (Table 3), and body weight recovered during treatment, indicating good overall tolerability. Given that current AML therapies are often limited by severe cardiac, hepatic, and bone marrow toxicity (Molica et al. 2024; Siaravas et al. 2025), these findings support further investigation of Arctigenin as a potentially low‐toxicity antileukemic candidate. Nevertheless, the present in vivo study evaluated Arctigenin at a single dose of 100 mg/kg and therefore does not establish its minimum effective dose or dose–response relationship. Further studies using multiple dose levels are required to determine the optimal therapeutic dose of Arctigenin.

## 5 Conclusion

This study is the first to reveal the antileukemic potential of ginger extracts. Through metabolomic screening followed by UHPLC–MS verification using an authentic standard, Arctigenin was identified as a promising antileukemic candidate compound from ginger. Integrated computational analyses and experimental observations suggest that PARP1 and NFκB1 may be involved in its antileukemic effects, while apoptosis induction was experimentally demonstrated. Furthermore, Arctigenin significantly suppressed tumor growth in vivo and exhibited a favorable safety profile under the conditions examined. These findings support Arctigenin as a promising plant‐derived candidate for further investigation in AML.

## Author Contributions

**Mengjun Huang:** investigation, methodology, funding acquisition, resources, writing – review and editing. **Jing Liu:** investigation, writing – original draft, writing – review and editing. **Kaixuan Zeng:** investigation, writing – original draft, writing – review and editing. **Lianling Xu:** investigation, writing – original draft, writing – review and editing. **Yangchun Liu:** investigation, writing – original draft, writing – review and editing. **Linyun Xie:** investigation, writing – original draft, writing – review and editing. **Mengni Shi:** investigation, writing – original draft, writing – review and editing. **Yan Zeng:** methodology, supervision, writing – review and editing. **Wenjun Liu:** conceptualization, methodology, funding acquisition, supervision, writing – review and editing. **Ling Guo:** conceptualization, investigation, methodology, funding acquisition, supervision, writing – original draft, writing – review and editing.

## Funding

This work was supported by the National Natural Science Foundation of China (82204574), Sichuan Science and Technology Program (2024YFFK0271), Sichuan Science and Technology Plan Joint Innovation Program (2022YFS0622‐A3), and the key project of Southwest Medical University clinical medicine special program (2025LCYXZX31, 2024LCYXZX10), the Project of Chongqing University of Arts and Sciences (R2026SK04).

## Conflicts of Interest

The authors declare no conflicts of interest.

## Supporting information


**Figure S1:** Expression of the 10 hub genes in AML patients versus healthy controls. Data were retrieved and analyzed from GEPIA (http://gepia.cancer‐pku.cn/) on July 2, 2025.
**Figure S2:** mRNA and protein expression levels of CCND1, HIF1A, HSP90AB1, NFκB1, and PARP1 across various cancer cell lines. Data were retrieved and analyzed from The Human Protein Atlas database (https://www.proteinatlas.org) on July 2, 2025.
**Figure S3:** Molecular docking of Arctigenin with hub targets. (A) PARP1; (B) NFκB1. Arctigenin is shown as magenta sticks. The reference PARP1 inhibitor is shown in yellow, and the NFκB1 inhibitor is shown in orange. Molecular docking was performed and visualized using PyMOL.
**Figure S4:** Molecular dynamics simulation (MDS) results for the Arctigenin‐NFκB1 complex. (A) RMSD values over simulation time. (B) Protein flexibility variations (RMSF) during simulation. (C) Hydrogen bond dynamics throughout the simulation. (D) Radius of gyration (Rg) curve. (E) Solvent‐accessible surface area (SASA) changes. (F) MMPBSA binding free energy analysis. (G, H) Two‐dimensional and three‐dimensional free energy landscape mappings.

## Data Availability

The original data presented in the study are included in the article or Supporting Information further inquiries can be directed to the corresponding authors.
