# Supplementary material for: Identification of Antileukemic Compound From Ginger by Integrating UHPLC–MS, Network Pharmacology, Molecular Docking, Molecular Dynamics Simulation, and In Vitro and In Vivo Experimental Validation
Source: Food Sci Nutr. 2026 Sep 16;14(9):e72379. doi: 10.1002/fsn3.72379 (PMC13583061; doi:10.1002/fsn3.72379)
Supplement: Supplementary file 1 [file FSN3-14-e72379-s001.docx]

**Identification of anti-leukemic compound from ginger by integrating UHPLC–MS, network pharmacology, molecular docking, molecular dynamics simulation and *in vitro* and *in vivo* experimental validation**

Mengjun Huang ^1,†^, Jing Liu ^2,†^, Kaixuan Zeng^3,†^, Lianling Xu^3^, Yangchun Liu^3^, Linyun Xie^3^, Mengni Shi^4^, Yan Zeng^2^, Wenjun Liu^2,*^, Ling Guo^2,3,*^

^1^ National-Local Joint Engineering Research Center for Innovative Targeted Drugs, Chongqing University of Arts and Sciences, Chongqing 402160, P.R. China.

^2^ Department of Pediatric hematologic Oncology and Respiratory, Children’s Medical Center, The Affiliated Hospital, Southwest Medical University, Sichuan Clinical Research Center for Birth Defects, Southwest Medical University, Luzhou, Sichuan 646000, China

^3^ Pediatrics Department, Southwest Medical University, Luzhou, Sichuan 646000 (P. R. China).

^4^ Department of Clinical Medicine, School of Clinical Medicine, Southwest Medical University, Luzhou, Sichuan 646000 (P. R. China).

^*^ Corresponding author: [wenjun_liu@swmu.edu.cn](mailto:wenjun_liu@swmu.edu.cn); [guoling@swmu.edu.cn](mailto:guoling@swmu.edu.cn)

^†^ These authors contributed equally to this work.

Figure S1. Expression of the ten hub genes in AML patients versus healthy controls. Data were retrieved and analyzed from GEPIA (<http://gepia.cancer-pku.cn/>) on July 2, 2025.

Figure S2 mRNA and protein expression levels of CCND1, HIF1A, HSP90AB1, NFκB1, and PARP1 across various cancer cell lines. Data were retrieved and analyzed from The Human Protein Atlas database (<https://www.proteinatlas.org>) on July 2, 2025.

Figure S3 Molecular docking of Arctigenin with hub targets. (A) PARP1; (B) NFκB1. Arctigenin is shown as magenta sticks. The reference PARP1 inhibitor is shown in yellow, and the NFκB1 inhibitor is shown in orange. Molecular docking was performed and visualized using PyMOL.

Figure S4 Molecular dynamics simulation (MDS) results for the Arctigenin-NFκB1 complex. (A) RMSD values over simulation time. (B) Protein flexibility variations (RMSF) during simulation. (C) Hydrogen bond dynamics throughout the simulation. (D) Radius of gyration (Rg) curve. (E) Solvent-accessible surface area (SASA) changes. (F) MMPBSA binding free energy analysis. (G-H) Two-dimensional and three-dimensional free energy landscape mappings.
